# A Regulated Double-Negative Feedback Decodes the Temporal Gradient of Input Stimulation in a Cell Signaling Network

**DOI:** 10.1371/journal.pone.0162153

**Published:** 2016-09-01

**Authors:** Sang-Min Park, Sung-Young Shin, Kwang-Hyun Cho

**Affiliations:** 1 Department of Bio and Brain Engineering, Korea Advanced Institute of Science and Technology (KAIST), 291 Daehak-ro, Yuseong-gu, Daejeon, 34141, Republic of Korea; 2 Department of Biochemistry and Molecular Biology, School of Biomedical Sciences, Monash University, Clayton, Victoria, 3800, Australia; 3 Biomedicine Discovery Institute, Monash University, Clayton, Victoria, 3800, Australia; Universitat Pompeu Fabra, SPAIN

## Abstract

Revealing the hidden mechanism of how cells sense and react to environmental signals has been a central question in cell biology. We focused on the rate of increase of stimulation, or temporal gradient, known to cause different responses of cells. We have investigated all possible three-node enzymatic networks and identified a network motif that robustly generates a transient or sustained response by acute or gradual stimulation, respectively. We also found that a regulated double-negative feedback within the motif is essential for the temporal gradient-sensitive switching. Our analysis highlights the essential structure and mechanism enabling cells to properly respond to dynamic environmental changes.

## Introduction

For survival, cells should continuously sense and process signals to make an appropriate decision under dynamically fluctuating cellular environments [1–3]. They encode biological information on the identity and quantity of a stimulus in different forms of patterns, for instance, amplitude, frequency, and duration of a stimulus [3, 4]. Such information is decoded and interpreted by specific signaling networks (or circuits) to generate a specific cellular response [3, 5, 6]. For example, the p53-Mdm2 network encodes the gamma radiation signaling in form of oscillatory dynamics of p53 while UV signal is encoded in sustained activation of p53 [3, 7]. The epidermal growth factor (ERK) pathway that encompasses the son of sevenless (SOS) -mediated negative feedback loop encodes EGF stimulation in form of a transient dynamics of ERK while nerve growth factor (NGF) stimulation is encoded in form of a sustained response of ERK by the protein kinase C (PKC)-mediated positive feedback loop [8, 9]. Such biological information encoded in dynamics of signaling molecules can be interpreted through many different types of molecular mechanisms. For example, Ca^2+^/calmodulin-dependent protein kinase II (CaMKII) and PKC are well known molecular machineries that decode oscillatory dynamics of cytoplasmic calcium [10, 11]. The incoherent feedforward loop that consists of ERK and c-Fos translates the transient and sustained dynamics to proliferation and differentiation, respectively [12].

Another important dynamic feature of signal that conveys biological information into cells is speed of signaling. In reality, a receptor on the cell surface can be immediately exposed to and activated by an acute increase in ligand concentration. Alternatively, as a result of its regulated secretion, cells may experience a gradual increase when a ligand is secreted from a distant source because it takes time to accumulate and reach a certain threshold level by the affinity of the receptor [13]. Several previous studies demonstrated that cells are capable of decoding the temporal rate of signaling. For example, Hodgkin's Type III excitable neuron fires for a step input (an abrupt increase of stimulation) but not a slow ramp input though these inputs have the same final level, named as slope sensitivity [14–16]. Such slope sensitivity was also found in auditory brainstem neurons, spinal cord neurons, and dopaminergic neurons [14, 17]. Another example was displayed by Young et al. who examined the environmental pathway using *Bacillus subtilis* [18]. Cells activated the response factor σ^B^ in instant increase of ethanol but not the slow increase. Nene *et al*. investigated the speed-dependent cellular decision making by considering a model circuit composed of two master regulators that inhibit each other (with self-activating mechanisms) and two input nodes receiving signals with different temporal gradients [19–21]. Note that the effect of the temporal gradient of a signal has been also widely studied in other fields. For example, in climate science and ecology, failure to adapt to rapid changes in environment was investigated, named as rate-dependent tipping or rate-induced bifurcation [22, 23]. The rate of change of the CO_2_ concentration in the atmosphere was also revealed affecting the Atlantic thermohaline circulation [24].

In the same vein, a recent experimental study clearly demonstrated how biological information can be encoded in the *temporal gradient* of the input signal [13]. Ji and coworkers demonstrated that when the brain-derived neurotrophic factor (BDNF) is applied to neuron cells in two modes of acute or gradual increase (at which the input signals reach their common steady-state concentration), the receptor activation (Tyrosine receptor kinase B, TrkB) generates quite distinct patterns; acute stimulation induces transient response and gradual response brought about gradual stimulation [13]. In other words, different cellular responses were delivered by different temporal gradients of the input signal. While the internalization of the surface TrkB could be suggested as a possible mechanism of the transient response of TrkB [13], up to now, a systematic study has not been carried out to elucidate the relationship between the signaling network structure, its information decoding capability, and input signal gradient.

To address this problem, we explored all possible topologies for a three-node enzymatic circuit and examined the capability to decode the temporal gradient of input stimulation. From a large-scale computational simulation, we identified an entangled positive and negative feedbacks (EPNF) network motif that can robustly realize differential responses to the temporal gradient of input stimulation. Central to this circuit’s signal processing capacity is an embedded double-negative feedback loop. Through dynamical analysis, we further revealed that the regulated double-negative feedback (RDNF) circuit performs the function of temporal gradient-sensitive switching. We suggest that this regulated double-negative feedback is a hidden design principle enabling cells to decode the information that is encoded in the temporal gradient of an input signal.

## Results

### Exploring network motif topologies

Cellular signaling networks (or signal transduction system) can be conceptually depicted as three major modules: an input module, a regulatory module, and an output module. The input module may correspond to the receptor on the cell surface that receives extracellular signals and transfer them to its downstream molecules. The regulatory module may encompass a set of signaling molecules that merge and process signals transferred from the receptor(s). It can also play diverse regulatory roles. The output module may include output molecules that induce specific target genes and activate other signaling proteins to generate an actual cellular response such as proliferation, differentiation and apoptosis. For example, the receptor tyrosine kinase (RTK) signaling pathway includes a broad range of receptors (e.g., Insulin-like growth factor receptor (IGFR), transforming growth factor receptor (TGFR), epidermal growth factor receptor (EGFR), and TrkB) to which growth hormones binds, such as IGF, TGF, EGF, and BDNF. Once the receptor is activated, the signal is transferred to their downstream pathway in many different ways such as binding, (de)phosphorylation, and translocation. These signals are further processed and regulated by their specific pathways (e.g., ERK, phosphoinositide 3-kinase (PI3K) and so on) and eventually activates their effector molecules (e.g., c-Myc for proliferation, B-cell lymphoma 2 (Bcl-2) for survival). However, the three main modules of signaling networks are complicatedly interlinked through crosstalks and feedback loops. For instance, Akt negatively regulates the RTK receptors through forkhead box O (FOXO) inhibition [25, 26]. Similar regulatory patterns of RTK activation also occur by mitogen-activated or extracellular signal–regulated protein kinase (MEK) inhibition, which is mediated through the transcription factor Myc [27]. Although detailed regulation of signaling networks are quite diverse and complicated, the RTK signaling network including TrkB can be functionally simplified to minimal models by applying a coarse-grained approach [28, 29] while preserving the essential functions. In addition, modularity has been proven to be a prevalent feature of network biology [30, 31]. Thus, for the purposes of simplicity and computability we reduced the complexity of the RTK signaling network to a generic three node network as shown in S1 Fig.

**Fig 1:**
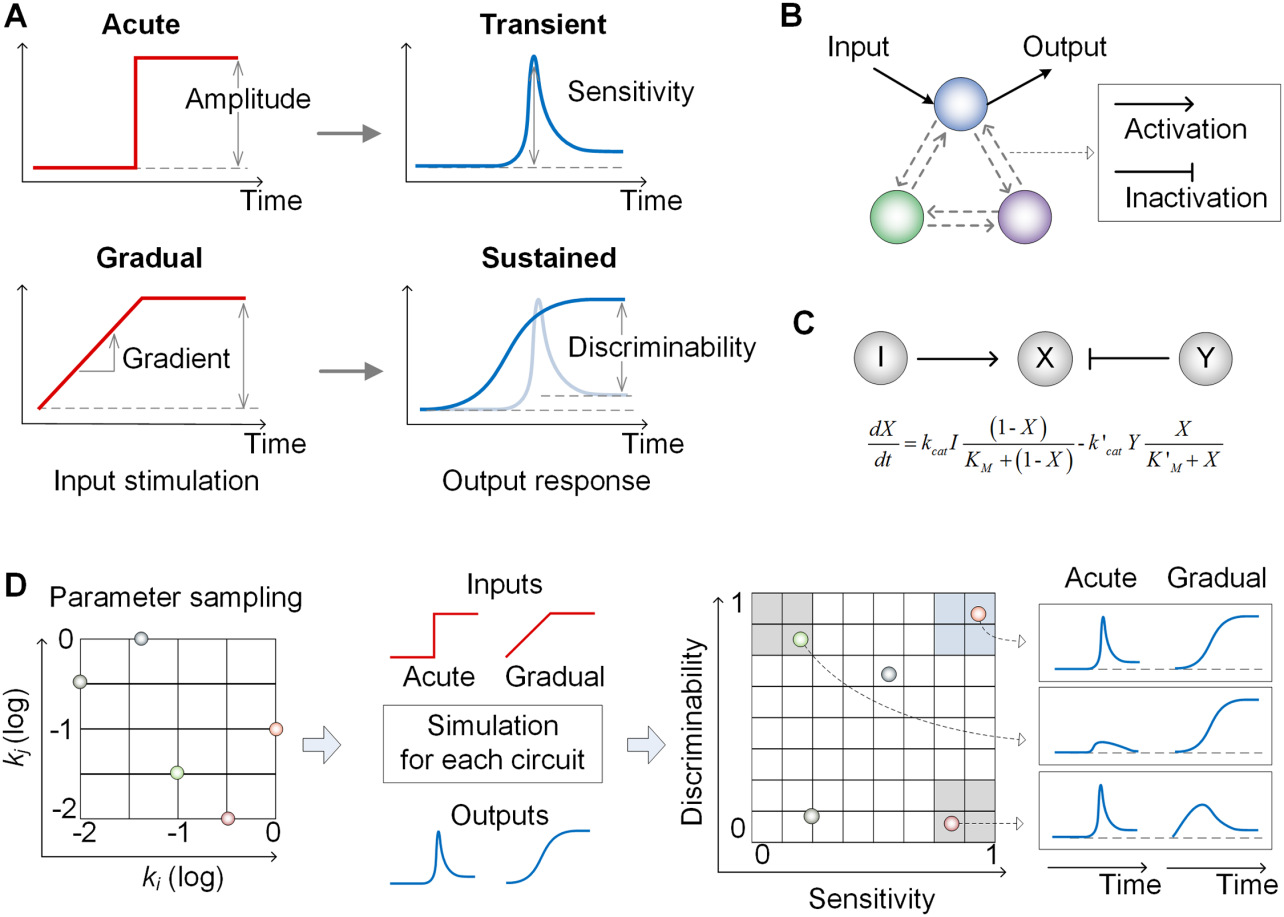
Overview of searching network topologies. **(A)** Targeted input-output relationship. Acute or gradual input stimulation causes transient or sustained output response, respectively. Discriminability and sensitivity are introduced as two criteria characterizing the dynamic behavior of a signaling network circuit. **(B)** Three-node network topology with all possible link combinations. **(C)** A simple example circuit modeled using Michaelis-Menten equation. **(D)** Illustration of the simulation of a candidate network circuit with sampled parameters and the evaluation of the resulting dynamic behavior with the two criteria.

As a result, we have limited ourselves to exploring three-node enzymatic networks that consist of input, regulatory, and output nodes corresponding to the three modules of the signaling network (Fig 1). Actually, this approach may sacrifice resolution. However, it allows us to efficiently perform a complete search of the topological space and to flexibly extract general design principles for specific cellular behaviors [32]. Note that to explore network motifs we considered the input receiving node as the output node of the network circuits (Fig 1B), following the experiment in which the activity of the BDNF-receiving TrkB receptor was measured as a readout of the signaling network [13]. Each link in the circuits can represent activation, inhibition or no regulation, and thus we generated 405 possible network topologies in total without symmetric cases. Each network topology was described using ordinary differential equations (ODEs) to model interactions of nodes, characterized by the Michaelis-Menten constants (KM’s) and catalytic rate constants (kcat’s) of the enzymes [32]. Note that in our analysis we implicitly assumed that the enzyme nodes operate under Michaelis-Menten kinetics and that they are noncooperative (Hill coefficient = 1) [32] (see Fig 1C, and Methods for details). We should also notice that it is widely accepted that the forms of rate equation (e.g., mass action and Michaelis-Menten equations) do not significantly change simulation results [32, 33] and thus we adopted Michaelis-Menten equations to formulate ODEs.

To address how the signaling network decodes the rate change of signals, we first defined the temporal gradient of the input signal as the increasing rate of the concentration of the stimulant and the amplitude as its steady-state concentration (Fig 1A). The targeted behavior of the network was characterized as a transient output (i.e., initial increase following decrease pattern) to the acute input, and a sustained output (i.e., monotonic increase pattern) to the gradual input. To quantitatively evaluate the responses profiles of the network topologies we introduced two criteria: (i) the discriminability of two outputs according to two different temporal gradients and (ii) the sensitivity of the transient output (Fig 1A, see Methods). The discriminability is defined as the differences of steady state values to acute and gradual inputs. The sensitivity is defined as the height of output response relative to the initial value.

In general, characterizing the dynamic behaviors of a network circuit requires intensive exploration of a large range parameter space of the model, and this is computationally exhausting. Furthermore, due to the sloppy nature of models in biology, it is difficult to analyze how robust the function of a model are with respect to the parameter changes [34]. Unfortunately, information on the sloppiness of the parameters is not generally available for our network model analysis, which means that all the parameters should be explored to determine the function of a motif circuit. Thus, to overcome the problems of sloppiness in the model and searching size of parameter space, 100,000 sets of parameters were efficiently sampled using the Latin hypercube sampling method [35] in a wide range of the nominal values from 0.01 to 10 fold (Fig 1D), and they were used to investigate how each circuit model decode the temporal gradient of the input signals.

### Identification of motif topology achieving the targeted behavior

**Fig 2:**
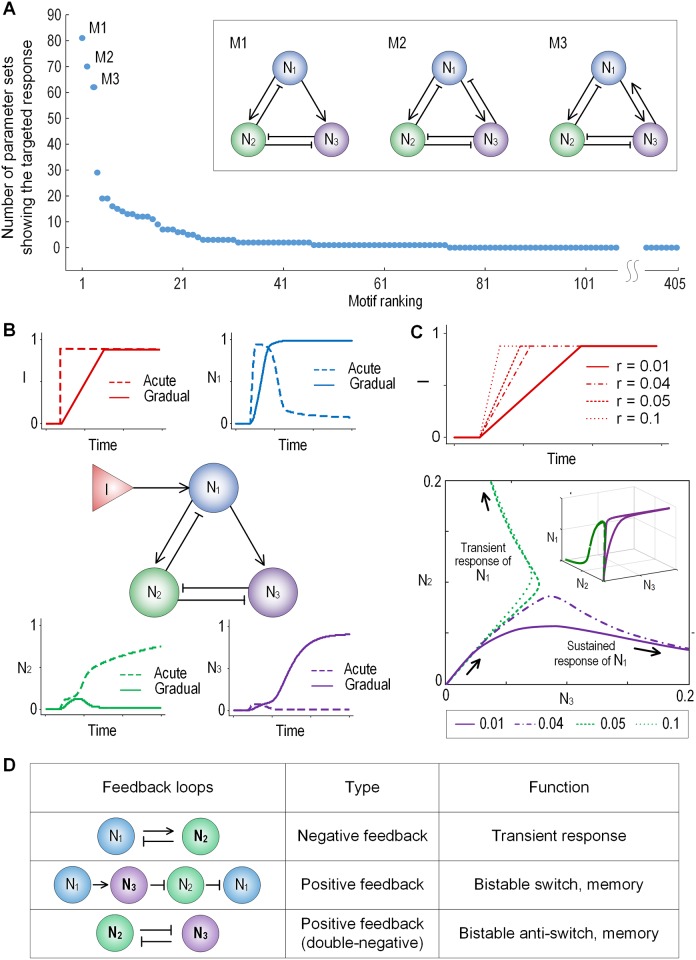
The input-output relationship and feedback structure of the EPNF circuit. **(A)** The number of parameter sets for all the network models. The parameter set was selected if the product of the sensitivity and the discriminability is more than 0.1. The most robust three motifs, (M1, M2, and M3) are shown in the inset. Note that M1 is EPNF, which is commonly contained in M2 and M3. **(B)** The representative response profiles of the EPNF network to acute and gradual increase of input stimulus, which recapitulate the predefined targeted behavior. **(C)** Typical input signal patterns with different temporal gradients (upper panel). The representative state trajectories of EPNF that were projected to the two dimensional space of N_2_ and N_3_ (lower panel **(D)** Three feedback loops comprising the EPNF circuit. The first two positive and negative feedback loops are interlinked at the output node N_1_ and these feedbacks are further controlled by the third double-negative feedback loop that might selectively turn on N_2_ or N_3_.

To identify the network motifs capable of decoding the temporal gradient of the input signal as shown in Fig 1, we generated 405 network motif circuits by considering all possible structures of a 3 node network without symmetric cases. Each network topology was simulated with 100,000 random parameter sets and the sensitivity and the discriminability of the output profile were calculated (Fig 1D). The response profile of each network topology can be mapped on the two-dimensional sensitivity and discriminability plot. Thus, a particular network circuit (and a specific parameter configuration) displaying the targeted behavior falls within the upper-right corner of the map. These circuits show a strong transient response (high sensitivity) to acute input signal and a strong sustained response (high discriminability) to the gradual signal. The network motif and number of parameter sets showing the target behavior was also plotted histogramatically in Fig 2A. From this plot, we identified three network circuits highly ranked in terms of number of parameter sets (M1, M2, and M3). Actually, the number of parameter sets corresponding to the highly ranked networks motif are more than twice that of other networks. Thus, we can say that these three network motifs are more ‘robust’ than other topologies also exhibiting the rate decoding capability. Here, we defined the robustness of a model by the number of parameter sets (of the 100,000 tested sets) that allow the model to generate targeted behavior [29]. We should note that if each parameter set of a network model represents a specific individual (e.g., cell) and the number of parameter set of a specific model is much larger than others, it could assume that this model population is evolutionally more robust.

Among the robust network topologies, we identified that the M1 motif was commonly shared in the three network circuits and it consists of a negative feedback loop between the node N_1_ and N_2_, a double-negative loop between N_2_ and N_3_, and a direct regulation link from N_1_ to N_3_ (Fig 2A). We named it as entangled positive and negative feedbacks (EPNF). Note that the lower ranked motifs, M4-M6 encompass a double-negative feedback that mutually inhibits N_1_ and N_2_ (S2 Fig). Thus, these networks were, by definition, not classified to EPNF. The representative response profiles of the EPNF to different input patterns (i.e., acute and gradual) were shown in Fig 2B. For the acute input, the node N_1_ displayed a transient response profile. N_2_ and N_3_ showed sustained and transient response curves, respectively. For the gradual input, N_1_ exhibited a sustained response. N_2_ and N_3_ showed a transient and sustained response, respectively. In the further analysis, we found that the sensitivity scores of the higher ranked motifs (i.e., M1-M3) showed relatively even distribution while the discriminability exhibited a biphasic distribution: both high (>10^−0.1^≈0.79) and marginal values (<10−0.9≈0.13) were dominant (S3 Fig). On the other hand, the sensitivity scores of the lower ranked motifs (M4-M6) showed relatively even distribution similar to the higher ranked motif (except M4 that was biased to the higher score). However, interestingly, the distribution of discriminability scores was strongly biased toward the lower score (<0.13), which suggests that the higher and lower ranked motifs have distinctive dynamic features. In summary, among the network motifs that are capable to decode the temporal gradient of the input signal, the EPNF-containing networks are more robust than other networks.

Next, we examined how the EPNF circuit decodes the temporal gradient of the input signals into different output responses. To do this, we introduced the gradient coefficient of the input signal (denoted by *r*) and gradually increased the steepness of the input signal (Fig 2C upper panel). Depending on the *r* value, trajectories of EPNF converged to different steady-states (Fig 2C lower panel). Interestingly, the trajectory curves in two dimensional space of N_2_ and N_3_ were bifurcated into two distinct steady-states. If the temporal gradient of the input is lower than the threshold value (less than 0.04 in this case), the trajectories proceed to stable states where N_2_ is low and N_3_ is high; otherwise, we got different stable states where N_2_ is high and N_3_ is low.

To further understand the underlying mechanism of the rate decoding and the bifurcation of trajectories, we dissected the EPNF motif circuit into a smaller piece of motifs. As shown earlier, it encompassed three feedback loops including a negative feedback, a positive feedback, and a double-negative feedback loop (Fig 2D). Such feedback loops in the signaling network have been known to have a specific role in generating different dynamic responses [36–39]. For example, a negative feedback can produce transient and oscillatory behaviors [36], and a positive feedback can generate a sustained response and exhibit a bistable switch-like response to make all-or-none responses [40]. Similarly, a double-negative feedback (functionally forming a positive feedback) can function as a bistable switch, enabling the systems to have two discrete, alternative stable steady states. [40–44]. In the EPNF structure, the node N_1_ mediates two feedback loops; one is the negative feedback through N_2_, and the other is the positive feedback through N_3_ and N_2_. Thus, if N_2_ is activated, the activation of N_1_ is suppressed by a negative regulation. If N_3_ is activated, N_1_ is sustainedly activated by a positive regulation. The activation states of N_2_ and N_3_ are mutually exclusive by the double-negative feedback loop between them, which suggests that this feedback loop is responsible for distinct response profiles of N_1_. Therefore, we presumed that the double-negative feedback loop embedded in the EPNF is a central mechanism that decodes the temporal gradient of the input signal.

### Regulated double-negative feedback is a gradient sensitive switch

**Fig 3:**
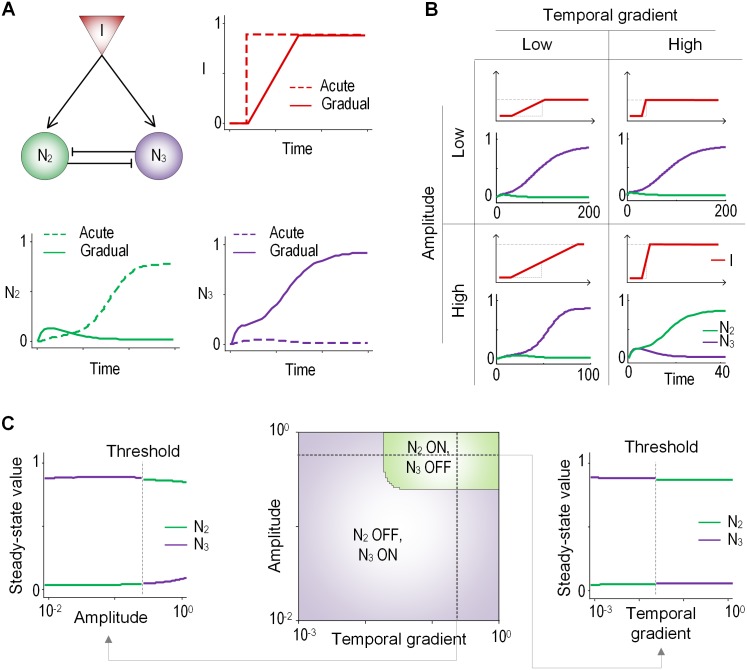
Bifurcation properties of the RDNF circuit. **(A)** The RDNF circuit and a representative response profile to two different temporal gradient of input. **(B)** Response patterns of N_2_ and N_3_ to different amplitude and temporal gradient. **(C)** Steady-state responses of N_2_ and N_3_ to various input amplitudes and temporal gradients. Bifurcation occurs depending on not only the input amplitude but also the temporal gradient.

To test whether the double-negative feedback loop of the EPNF is enough to decode the temporal gradient of the input signal, we further simplified the EPNF motif to the two-node network topology that consists of N_2_ and N_3_ nodes, and one input (I) which activates N2 and N3 at same time. We called it the regulated double-negative feedback (RDNF). The simulation results showed that N_2_ and N_3_ have opposite response profiles to the two different input patterns (Fig 3A). That is, N_2_ and N_3_ displayed a sustained and a transient response, respectively, to acute stimulation, but the response profiles to gradual stimulation were switched with each other, which suggests that the RDNF itself functions as a rate decoder of the temporal gradient of the input signal.

In previous studies the RDNF motif was suggested to have capability to decode the change in the amplitude of the input signal [43, 45], although our simulation results showed the decoding capacity of the temporal gradient. Thus, we carried out the additional simulation to analyze the effect of the amplitude of the signal on the rate decoding capability of the signaling network. From the simulation analysis the opposite response profiles (sustained and transient) were showed in N_2_ and N_3_, respectively, to the high gradient and high amplitude input signals, and the response patterns were switched by the low gradient with the same amplitude (Fig 3B). In contrast, the two nodes exhibited the opposite output responses to low amplitude and high gradient but the pattern were not switched by low gradient with the same amplitude. These observations prompted us to further simulate the response profiles of the RDNF by gradually increasing the amplitudes and temporal gradients of the input signal. We plotted two dimensional heat map of amplitude and gradient (Fig 3C middle). From this figure, we identified a sharp transition of the response profiles depending on the temporal gradient as well as the amplitude of the input (Fig 3C left and right). The steady state value of N_2_ (N_3_) were sharply switched from low (high) to high (low) around the threshold level of the amplitude and gradient. Taken together, these results hypothesize that the RDNF is able to decode two different dynamic features (i.e., the amplitude and the temporal gradient) of the input stimulation.

### Phase plane analysis of the temporal gradient decoding

**Fig 4:**
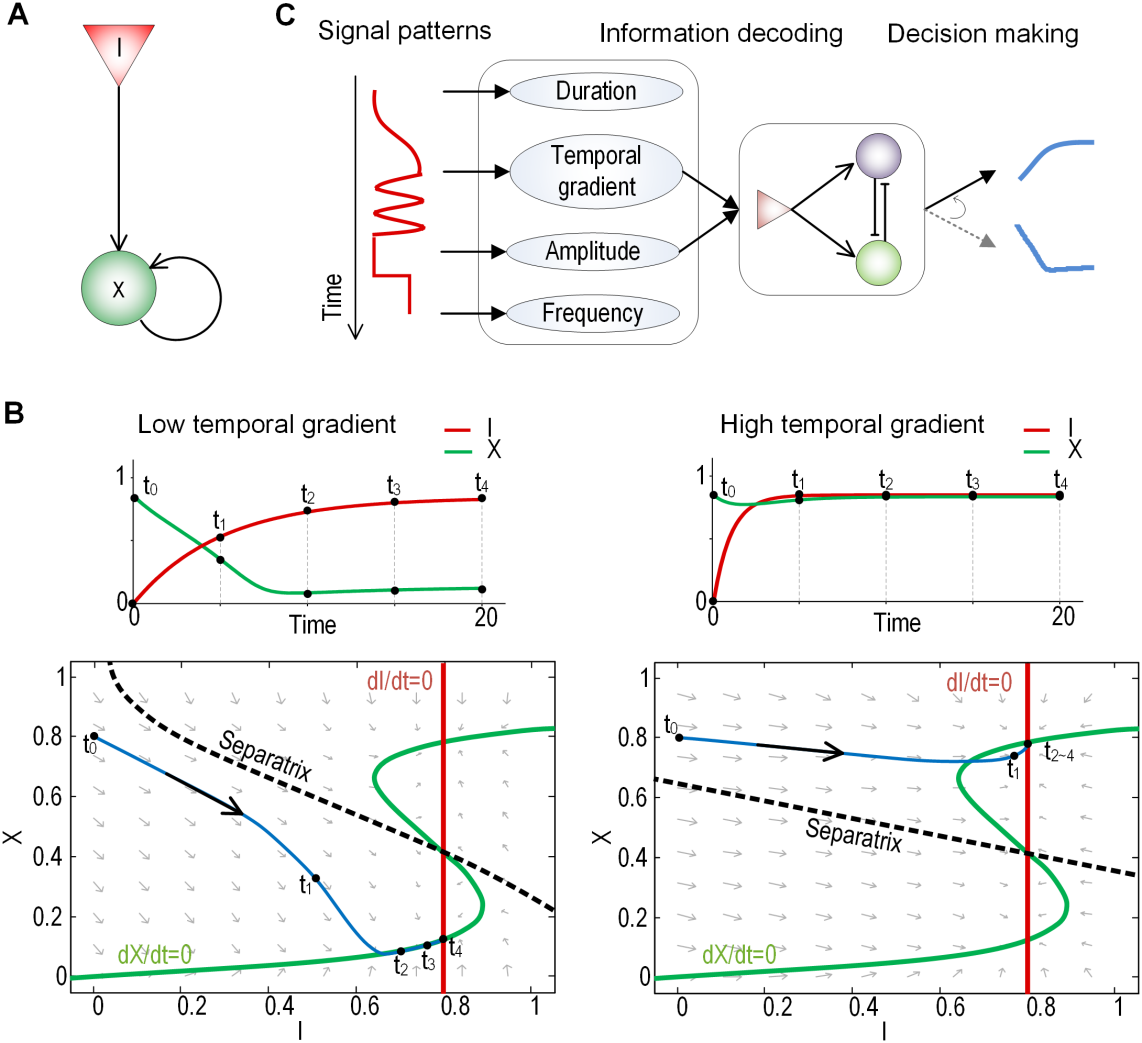
Analysis of the temporal gradient decoding mechanism. **(A)** A simplified single node network. **(B)** Input-output relationship (top) and phase plane analysis (bottom) of the simplified model with two different temporal gradients. The temporal gradient influences the state trajectories and determines the critical state transition towards different steady-states. **(C)** Schematic illustration of the cellular signal decoding process by the proposed temporal gradient-sensitive switch network.

A double-negative feedback can be functionally simplified to a single positive feedback since both the double-negative feedback loop and the positive-feedback loop can convert a graded input into switch-like, irreversible responses [40]. Thus, to visualize the dynamic behaviors of the network and to analyze the rate decoding mechanism more intuitively we further simplified the RDNF motif to an one node network topology composed of two variables, one input (I) and one output (X) with positive autoregulation as shown in Fig 4A, since a system with more than three dimensions like the RDNF makes it difficult to analyze the dynamic behaviors based on the phase-plane method. Note that the input variable *I* of the reduced network is a continuous function with the temporal gradient *r* and the maximal amplitude *I*_*0*_ (see Methods for model equations) and thus the dynamic behaviors of the network topology is displayed in a two dimensional phase plane.

The simulation results showed that a single node positive feedback loop can generate different response patterns to different temporal gradients. We found that the response converges to a low steady state value by a low temporal gradient but to a high steady state by a high gradient of the input signal (Fig 4B, upper panel). The phase plan clearly demonstrated the input-output dynamics of the reduced system (Fig 4B bottom). The nullclines of the system (*dI/dt* = 0 and *dX/dt* = 0, respectively) drawn in the phase plane were intersected at three fixed points, in which two are stable and one is an unstable point. It means that all solution trajectories for the given initial values eventually converge to one of the stable fixed points (called attractors) which correspond to steady-state responses. A set of initial conditions leads to trajectories that converge to a particular stable fixed point and we call it the basin of attraction. In this simulation we found that the separatrix of the basins (boundary between basins) was changed by different temporal gradients, which means that even the solution trajectories starting at the same initial points can be converged at different fixed points depending on the temporal gradient values. For example, the solution trajectories starting at the same initial values (i.e., (X, I) = (0.8, 0)) are moving along different pathways and eventually approach different stable fixed points (Fig 4B bottom). In other words, by changing the territory of the basin, the network may decode the temporal gradient. However, we note that the maximum amplitude of the input (*I*_*0*_) influences the nullcline locations of the input and, thus, the existence of bistabiliy may be determined by strength of input signal (S4 Fig). To further validate these findings, we carried out additional simulation using the more complex EPNF network. To do this, we changed the temporal rate and amplitude of the input signal and observed the trajectories of N_2_ and N_3_. Interestingly, the sepatrix of the basins was changed by different rates (S5A–S5C Fig left and middle). That is, the trajectories starting at the same initial states were converged to different attractors depending on the temporal gradient while the attractors remained unchanged. On the other hand, the location of attractors was moved to different places by changing of the amplitude (S5C Fig middle and right). Considering all the simulation results, we conclude that the amplitude of the input signal is related to the location of fixed points and that the temporal gradient determines the fixed points to which the rate decoding system converges.

## Discussion

How the signaling network encodes and decodes the biological information and what mechanism underlies this process have been a fundamental question in biology [1–3, 37]. Our study revealed that the EPNF topology decode the speed change of the input signal into two different response profiles of sustained and transient. In addition, we also showed that the EPNF was enough to recapitulate the rate decoding capacity of TrkB signaling network to BDNF stimulation [13]. In detailed analysis, we identified the RDNF topology, a subset of the EPNF, is a core mechanism of the rate decoding function. Although motivated by the study of Ji et al [18] in which the input receiving node was considered as the output node of the network circuits, our findings demonstrated that the rate-decoding capacity of the network do not depend on the choice of input/output since N_2_ and N_3_ clearly exhibited distinct response profiles to two different input gradients (Fig 2B). Moreover, the fact that the regulated double-negative feedback is ultimate underlying mechanism of the rate decoding of the input signal (Fig 3A) definitely supports that the rate decoding capability of the network does not depend on the choice of an output node. The phase plane analysis explained that the temporal gradient of an input signal determines the direction of the trajectory (solution) curves which leads to different steady-states of the system. In summary, our findings bring a new insight into how cells interpret the time-varying cellular environment which they might continuously encounter.

Under fluctuating cellular environments, the signal transduction system might have evolved to decode not only the steady-state level of input stimulation but also its temporal pattern for a proper decision-making (Fig 4C). Decoding the temporal gradient of input stimulation provides cells with additional useful information about the environment. For example, akin to the manner in which a safety belt senses acceleration, the temporal gradient of a car’s speed, cells can detect a rapid increase of a toxic molecule’s concentration and cope with it by activating a protection pathway in advance. In addition, cells can respond appropriately to a constitutive or regulated secretory mode of molecules by decoding the temporal gradient, as argued previously [13]. For a further study, it would be interesting to investigate the possibility of the temporal gradient decoding mechanism with respect to cellular recognition of the frequency of an oscillating signal.

Previous studies characterized the RDNF circuit that can be employed for decoding morphogen gradients to select gene activation in response to different levels of a single external signal and buffering their profile against fluctuations in gene dosage or environmental perturbations [43, 46]. For instance, Saka et al. demonstrated that the mutual inhibitory genetic network having a single input can convert a graded signal into an on/off binary output [43]. Bergman *et al*. modeled and analyzed that an interlinked multiple negative feedback mechanism is capable of pre-steady-state decoding a spatial gradient signal to expression of different target genes [46]. In this study, we showed that the double-negative feedback network that was derived from the EPNF can interpret the rate change of an input signal to different response profiles of target proteins before the signal converge to a steady state. Thus, we can infer that the RDNF circuit might have a crucial role in early differentiation during development since the double-negative feedback loop may interpret information regarding the temporal dynamics of morphogen fluctuation to elaborately decide the cell fate [43, 47]. Taken together the double-negative feedback mechanism can be obtained in different ways in different biological contexts, and it can provide a general mechanism for controlling the cell-fate decision.

From this study, we proposed that the cell can respond differently according to the temporal pattern of stimulation even though the final concentrations are unchanged, which suggests that the delivery mode is important in determining the effect of the molecules.

## Methods

### Candidate motif circuit topologies

To find the motif circuit for decoding the temporal gradient of input stimulation, we search possible topologies of three-node enzymatic regulatory networks. Within a motif circuit, there are six possible directed links among the nodes. Each link has three possibilities: positive regulation (activation), negative regulation (inhibition), or no regulation. Thus there are 3^6^ = 729 possible topologies. By excluding circuits with symmetric cases, the remaining 405 topologies are explored in this study. An input receiving node of the motif circuits is regarded as an output responding node to mimic the BNDN experiment [13].

### Modeling motif circuits

Each node of a motif circuit can have an active or inactive form, which is reversible by positive or negative regulation, and has a fixed total concentration of 1 by normalization. In the modeling scheme, we assume that a catalytic reaction of the enzymatic network consists of forward and backward reactions. The forward reaction indicates that the active enzyme (E, for example), convert the other enzyme (X) from its inactive to active state (see the rate equation below). The backward reaction is, inversely, that the active enzyme (X) is converted to inactive state by another active enzyme (Y). If the enzyme that catalyzes the forward or backward reaction is not specified in the network, we assume that a basal (nonregulated, unspecific) enzyme would activate of inactivate X, respectively. All regulations of a motif were modeled with Michaelis-Menten equations (S1 Text). All parameters in the equations were sampled from a range of 0.01 to 10. The general equation for a motif is modeled combining the forward and the backward reactions as follows:

$$\frac{dN_{i}}{dt}=\sum_{l}k_{N_{l}N_{i}}N_{l}\frac{\left(1-N_{i}\right)}{K_{N_{l}N_{i}}+\left(1-N_{i}\right)}-\sum_{m}k\prime_{N_{m}N_{i}}N_{m}\frac{N_{i}}{K\prime_{N_{m}N_{i}}+N_{i}},$$

where *N*_*i*_ = *N*_*1*_, *N*_*2*_ or *N*_*3*_ denote the normalized concentration of active form of nodes; 1—*N*_*i*_ denote the concentration of inactive form of nodes; *N*_*l*_ = *N*_*1*_, *N*_*2*_, *N*_*3*_ is *I* (input) or *E* (active enzyme) that has a positive regulation to node N_i_; *N*_*m*_ = *N*_*1*_, *N*_*2*_, *N*_*3*_ or *E* denote the concentration of the node which has a negative regulation to node N_i_; $K_{N_{l}N_{i}}$ or $K\prime_{N_{m}N_{i}}$ denote the Michaelis-Menten constants, and $k_{N_{l}N_{i}}$ or $k\prime_{N_{m}N_{i}}$ denote the catalytic rate constants of the regulations. All the equations of the analyzed motifs and parameters used in the figures are provided in S2 Text.

### Evaluation of network motifs

The input signal *I* is modeled as follows:

$$\frac{dI}{dt}=\{\begin{array}{c} r\\ \begin{array}{l} 0 \end{array} \end{array}\;\;\;\;\;\;\begin{array}{c} \left(t<\frac{I_{0}}{r}\right)\\ \left(t\geq \frac{I_{0}}{r}\right) \end{array}$$

where *I*_0_ denotes the maximum amplitude of the input signal and *r* denotes the temporal gradient. Note that only increasing input is considered in our simulations. In the case of an acute input, *r* is regarded as having an infinite value. The network motifs were simulated with inputs after the response profile converges to a steady-state.

The dynamic behavior of a simulated circuit model was evaluated by the discriminability and sensitivity, defined as follows:

$$\begin{array}{l} Sensitivity=\;M_{a}-F_{a}\\ Discriminability\;=\;F_{g}-F_{a} \end{array}$$

where *F*_*a*_ and *F*_*g*_ denotes the final values of the output response profiles to acute or gradual inputs, respectively, and *M*_*a*_ denotes the maximum value of the output response to acute input. The capability of a network motif was measured by the score of the sensitivity and the discriminability. If the product of sensitivity and discriminability is more than 0.1, the network circuit is defined to be able to decode the temporal gradient.

### Dynamical analysis of the simplified model

The mathematical description of the simplified model (Fig 4A) is as follows:

$$\begin{array}{l} \frac{dI}{dt}=r\left(I_{0}-I\right)\\ \frac{dX}{dt}=\left(1-X\right)\frac{k_{Xa}\times I}{K_{Xa}+\left(1-X\right)}+\left(1-X\right)\frac{k_{Xb}\times X}{K_{Xb}+\left(1-X\right)}-X\frac{k_{Xc}}{K_{Xc}+X} \end{array}$$

where *I* denotes exponentially increasing input, *I*_0_ denotes the maximum amplitude, *r* denotes the temporal gradient, and *X* denotes the positively self-regulating node.

Phase plane illustrates all the states and trajectories in the state space. The nullcline is a curve where the derivative of a system variable equals zero. The fixed points are located on intersecting points between nullcline curves of a system. According to the stability of the points, they are categorized as stable fixed points and unstable fixed points. All the states of the system consequently approach one of the stable points.

## Supporting Information

S1 Fig Modeling of the three-node enzymatic network. The RTK signaling networks can be conceptually depicted as three major modules: an input module, a regulatory module, and an output module. This signaling network can be functionally further simplified to minimal models by applying a coarse-grained approach while preserving the essential functions. In addition, modularity has been proven to be a prevalent feature of network biology. Thus, for the purposes of simplicity and computability the complexity of the original model was reduced to a generic three-node enzymatic network.
(TIF)

S2 Fig Six network motifs ranked by robustness to exhibit the targeted behaviors. M1-M3 are the high-ranked motifs and M4-M6 are the low-ranked motifs.
(TIF)

S3 Fig Distribution of the sensitivity and the discriminability of the six network motifs. Sensitivity and discriminability from the simulation of parameter sets are represented in the scatter plot with marginal histogram. X-and Y-Axis are log-scale, respectively.
(TIF)

S4 Fig Effect of the amplitude of the input. Input-output relationship (top) and phase plane analysis (bottom) of the simplified model with the changed amplitude of the input *I*_*0*_ from Fig 4B. *I*_*0*_ affected the nullcline location of the input and determined the existence of bistabiliy. When *I*_*0*_ is 0.5, only one stable fixed point exists.
(TIF)

S5 Fig Different effect of the temporal gradient and the amplitude of the input signal on the basin of attractors of the EPNF network. **(A)** Blue or red regions are the basin of attractors which correspond to transient or sustained responses of N_1_, respectively. The inset shows the basin of attractors in full-scale and the blue and red circle are attractors. **(B)** Sample state trajectories starting at the different initial states, which are projected in two dimensional space of N_2_ and N_3_. Blue or red lines corresponds to the basins of (A). **(C)** Sample state trajectories starting at the different initial states in three dimensional space of N_1_, N_2_ and N_3_. The location of attractor is noted as (N_1_, N_2_, N_3_).
(TIF)

S1 Text Derivation of the equations for modeling the motif circuits. (DOCX)

S2 Text Equations of the analyzed motifs and mathematical kinetic parameters used in figures. (DOCX)
