# Supplementary figures and images for: A Regulated Double-Negative Feedback Decodes the Temporal Gradient of Input Stimulation in a Cell Signaling Network
Source: PLoS One. 2016 Sep 1;11(9):e0162153. doi: 10.1371/journal.pone.0162153 (PMC5008701; doi:10.1371/journal.pone.0162153)

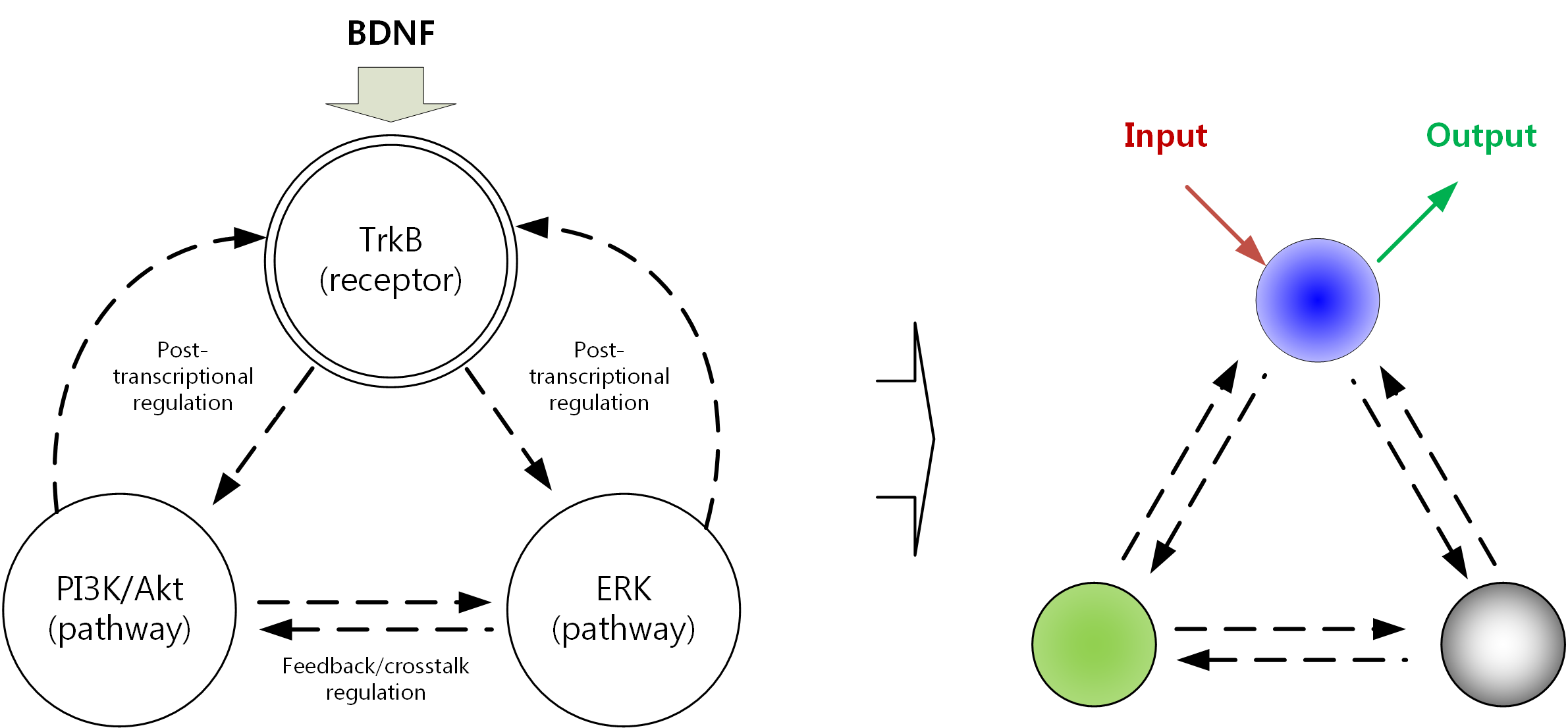

Supplement: S1 Fig — The RTK signaling networks can be conceptually depicted as three major modules: an input module, a regulatory module, and an output module. This signaling network can be functionally further simplified to minimal models by applying a coarse-grained approach while preserving the essential functions. In addition, modularity has been proven to be a prevalent feature of network biology. Thus, for the purposes of simplicity and computability the complexity of the original model was reduced to a generic three-node enzymatic network. (TIF) [file pone.0162153.s001.tif]

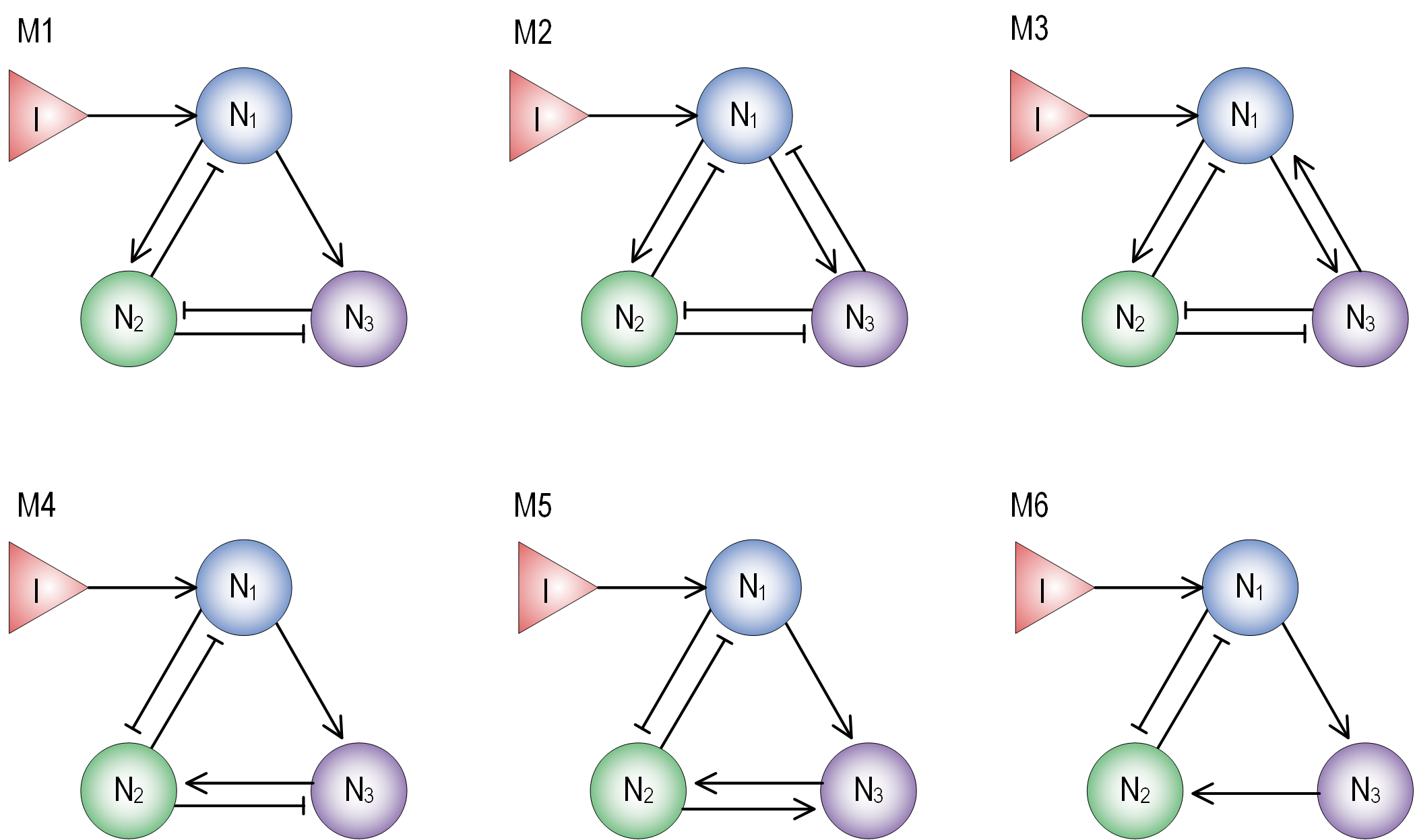

Supplement: S2 Fig — M1-M3 are the high-ranked motifs and M4-M6 are the low-ranked motifs. (TIF) [file pone.0162153.s002.tif]

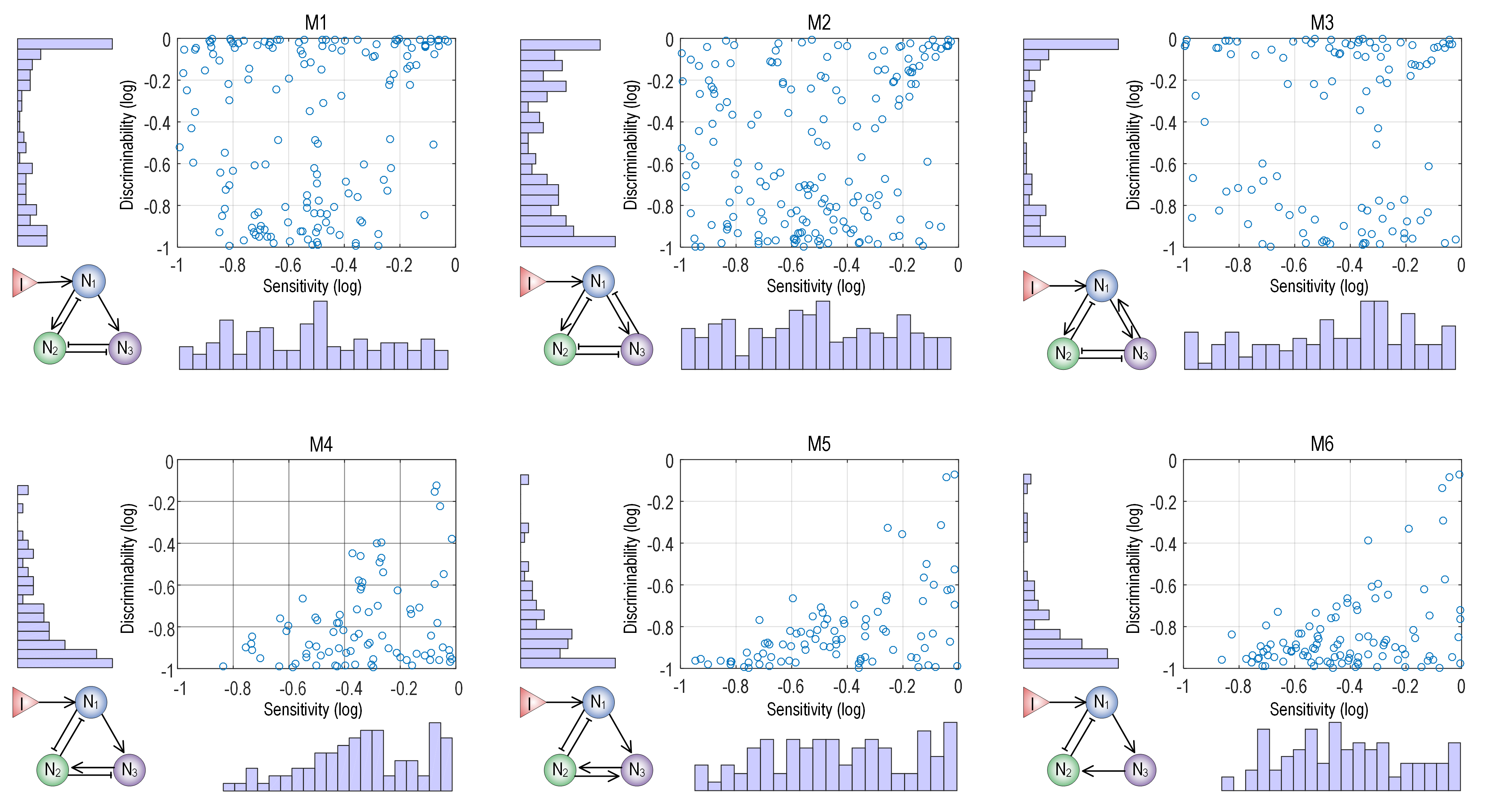

Supplement: S3 Fig — Sensitivity and discriminability from the simulation of parameter sets are represented in the scatter plot with marginal histogram. X-and Y-Axis are log-scale, respectively. (TIF) [file pone.0162153.s003.tif]

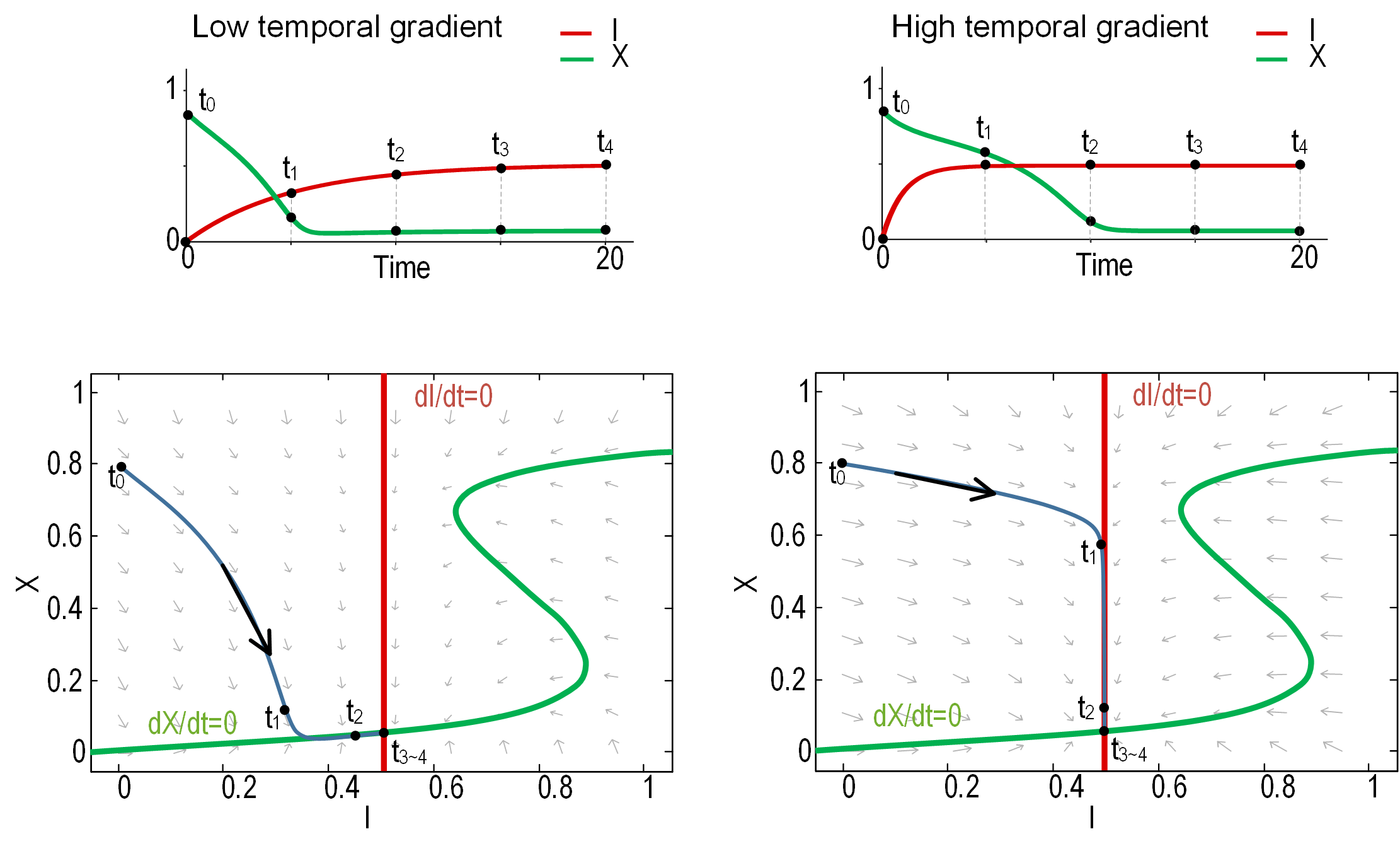

Supplement: S4 Fig — Input-output relationship (top) and phase plane analysis (bottom) of the simplified model with the changed amplitude of the input I0 from Fig 4B. I0 affected the nullcline location of the input and determined the existence of bistabiliy. When I0 is 0.5, only one stable fixed point exists. (TIF) [file pone.0162153.s004.tif]

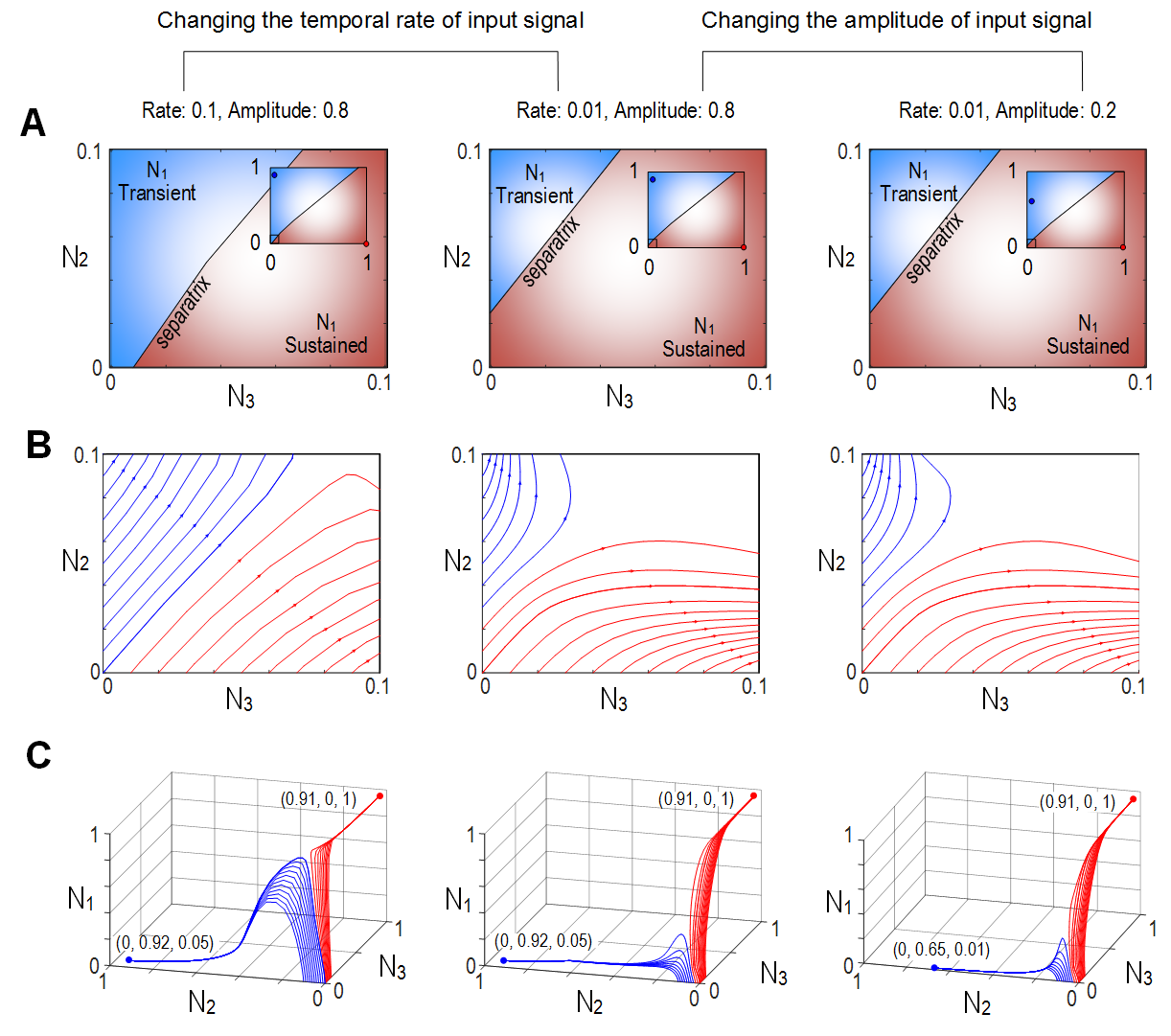

Supplement: S5 Fig — (A) Blue or red regions are the basin of attractors which correspond to transient or sustained responses of N1, respectively. The inset shows the basin of attractors in full-scale and the blue and red circle are attractors. (B) Sample state trajectories starting at the different initial states, which are projected in two dimensional space of N2 and N3. Blue or red lines corresponds to the basins of (A). (C) Sample state trajectories starting at the different initial states in three dimensional space of N1, N2 and N3. The location of attractor is noted as (N1, N2, N3). (TIF) [file pone.0162153.s005.tif]
