# Supplementary material for: A Regulated Double-Negative Feedback Decodes the Temporal Gradient of Input Stimulation in a Cell Signaling Network
Source: PLoS One. 2016 Sep 1;11(9):e0162153. doi: 10.1371/journal.pone.0162153 (PMC5008701; doi:10.1371/journal.pone.0162153)
Supplement: S1 Text — (DOCX) [file pone.0162153.s006.docx]

**S1 Text. Derivation of the equations for modeling the motif circuits.**

The formula of the rate equation employed in this study was derived from of the typical Michaelis-Menten equation by assuming that the concentration of the reactant is conserved and the total amount of substrate is much larger than enzyme ($[S]_{0}\gg[E]_{0}$). For example, the schematic diagram and the rate equation of the typical Michaelis-Menten equation are expressed as follows:

,

$v=\frac{d[P]}{dt}=\frac{V_{\max}[S]}{K_{M}+[S]}$, $K_{M}=\frac{k_{-1}}{k_{1}}$ and $V_{\max}=k_{2}[E_{1}]_{0}$

where [S] and [P] denote the concentration of the substrate and the product, respectively. $[E]_{0}$ denotes the total concentration of the enzyme. In the modeling of the signaling pathway, we call this reaction ‘forward reaction’ since the product is generated from the substrate. Thus, the schematic diagram of the forward reaction can be simply drawn as follows:

.

In the biochemical reaction, the reverse reaction is also possible. That is, the product is converted to the substrate by another type of enzyme; we call this reaction ‘backward (or reverse) reaction’ and the schematic diagram and the rate equation can be described as follows:

,

$v=\frac{d[\mathrm{S}]}{dt}=\frac{V'_{\max}[\mathrm{P}]}{K'_{M}+[\mathrm{P}]}$, where $V'_{\max}=k'_{2}[\mathrm{E}_{2}]_{0}$.

Combining the forward and the backward reaction together, we obtained the following rate equations:

,

$v=\frac{d[P]}{dt}=\frac{V_{\max}[S]}{K_{M}+[S]}-\frac{V'_{\max}[\mathrm{P}]}{K'_{M}+[\mathrm{P}]}$.

Since the total concentration of the reactant is assumed to be conserved ($[S]_{0}=[\mathrm{S}]+[\mathrm{P}]$), we have

$v=\frac{d[P]}{dt}=\frac{V_{\max}\left([S]_{0}-[\mathrm{P}]\right)}{K_{M}+\left([S]_{0}-[\mathrm{P}]\right)}-\frac{V'_{\max}[\mathrm{P}]}{K'_{M}+[\mathrm{P}]}$.

Normalizing the rate equation by the total concentration of the substrate, we can obtain the following rate equation:

$v=\frac{d[P]}{dt}=\frac{V_{\max}\left(1-[\mathrm{P}]\right)}{K_{M}+\left(1-[\mathrm{P}]\right)}-\frac{V'_{\max}[\mathrm{P}]}{K'_{M}+[\mathrm{P}]}$.

Let P be an active form of the reactant ($X$) and S be an inactive form ($X_{inactive}$). The reaction scheme and the rate equation of example network in Fig. 1C are described as follows:

,

$\frac{dX}{dt}=k_{cat}I\frac{\left(1-X\right)}{K_{M}+\left(1-X\right)}-k'_{cat}Y\frac{X}{K'_{M}+X}$,

where $I$is an enzyme catalyzing the forward reaction and $Y$is the other enzyme that catalyzes the backward reaction.

In the case of a node in a network have only positive or negative regulation, which means that the enzyme that catalyzes the forward or backward reaction is not specified in the network, we assume that there is a basal (nonregulated, unspecific) enzyme out of a circuit has the rest regulation to the node. For example, if node X is negatively regulated by node Y and there is no positive regulation to node X in a network, the equation for node X is as follows:

$$\frac{dX}{dt}=k_{EX}\times E\frac{\left(1-X\right)}{K_{EX}+\left(1-X\right)}-k_{YX}\times Y\frac{X}{K_{YX}+X}$$

where E is a basal enzyme which has positive regulation to node X and whose concentration is fixed.
