# Supplementary material for: A Regulated Double-Negative Feedback Decodes the Temporal Gradient of Input Stimulation in a Cell Signaling Network
Source: PLoS One. 2016 Sep 1;11(9):e0162153. doi: 10.1371/journal.pone.0162153 (PMC5008701; doi:10.1371/journal.pone.0162153)
Supplement: S2 Text — (DOCX) [file pone.0162153.s007.docx]

**S2 Text. Equations of the analyzed motifs and mathematical kinetic parameters used in figures.**

Equations for the entangled positive and negative feedbacks (EPNF) in Fig. 2A are as follows:

$$\begin{matrix} \frac{dI}{dt}=\left\{\begin{matrix} r \\ \begin{matrix} \\ 0 \end{matrix} \end{matrix}\right. \begin{matrix} (t<\frac{I_{0}}{r}) \\ (t\geq\frac{I_{0}}{r}) \end{matrix} \\ \frac{dN_{1}}{dt}=k_{IN_{1}}\times I\times\frac{\left(1-N_{1}\right)}{K_{IN_{1}}+\left(1-N_{1}\right)}-k_{N_{2}N_{1}}\times N_{2}\times\frac{N_{1}}{K_{N_{2}N_{1}}+N_{1}} \\ \frac{dN_{2}}{dt}=k_{N_{1}N_{2}}\times N_{1}\times\frac{\left(1-N_{2}\right)}{K_{N_{1}N_{2}}+\left(1-N_{2}\right)}-k_{N_{3}N_{2}}\times N_{3}\times\frac{N_{2}}{K_{N_{3}N_{2}}+N_{2}} \\ \frac{dN_{3}}{dt}=k_{N_{1}N_{3}}\times N_{1}\times\frac{\left(1-N_{3}\right)}{K_{N_{1}N_{3}}+\left(1-N_{3}\right)}-k_{N_{2}N_{3}}\times N_{2}\times\frac{N_{3}}{K_{N_{2}N_{3}}+N_{3}} \end{matrix}$$

**Table A. Mathematical kinetic parameters for the entangled positive and negative feedbacks (EPNF) used in Fig. 2.**

| **Parameter** | **Value** | **Unit** |
| --- | --- | --- |
| *I_0_* | 8.000E-01 | [level] |
| *r* | 4.000E-02 | [level]·hour^-1^ |
| $k_{IN_{1}}$ | 1.798E+00 | [level]·hour^-1^ |
| $K_{IN_{1}}$ | 1.221E-01 | [level] |
| $k_{N_{2}N_{1}}$ | 4.994E+00 | [level]·hour^-1^ |
| $K_{N_{2}N_{1}}$ | 1.262E-01 | [level] |
| $k_{N_{1}N_{2}}$ | 3.498E-01 | [level]·hour^-1^ |
| $K_{N_{1}N_{2}}$ | 6.873E-01 | [level] |
| $k_{N_{3}N_{2}}$ | 5.315E+00 | [level]·hour^-1^ |
| $K_{N_{3}N_{2}}$ | 1.425E-01 | [level] |
| $k_{N_{1}N_{3}}$ | 2.326E-01 | [level]·hour^-1^ |
| $K_{N_{1}N_{3}}$ | 6.821E-01 | [level] |
| $k_{N_{2}N_{3}}$ | 5.916E+00 | [level]·hour^-1^ |
| $K_{N_{2}N_{3}}$ | 3.107E-01 | [level] |

Equations for the regulated double-negative feedback (RDNF) in Fig. 3A are as follows:

$$\begin{matrix} \frac{dI}{dt}=\left\{\begin{matrix} r \\ \begin{matrix} \\ 0 \end{matrix} \end{matrix}\right. \begin{matrix} (t<\frac{I_{0}}{r}) \\ (t\geq\frac{I_{0}}{r}) \end{matrix} \\ \frac{dN_{2}}{dt}=k_{N_{1}N_{2}}\times I\times\frac{\left(1-N_{2}\right)}{K_{N_{1}N_{2}}+\left(1-N_{2}\right)}-k_{N_{3}N_{2}}\times N_{3}\times\frac{N_{2}}{K_{N_{3}N_{2}}+N_{2}} \\ \frac{dN_{3}}{dt}=k_{N_{1}N_{3}}\times I\times\frac{\left(1-N_{3}\right)}{K_{N_{1}N_{3}}+\left(1-N_{3}\right)}-k_{N_{2}N_{3}}\times N_{2}\times\frac{N_{3}}{K_{N_{2}N_{3}}+N_{3}} \end{matrix}$$

**Table B. Mathematical kinetic parameters for the regulated double-negative feedback (RDNF) used in Fig. 3.**

| **Parameter** | **Value** | **Unit** |
| --- | --- | --- |
| *I_0_* | 8.000E-01 | [level] |
| *r* | 1.000E-02 | [level]·hour^-1^ |
| $k_{N_{1}N_{2}}$ | 9.139E-01 | [level]·hour^-1^ |
| $K_{N_{1}N_{2}}$ | 1.769E+00 | [level] |
| $k_{N_{3}N_{2}}$ | 1.672E+00 | [level]·hour^-1^ |
| $K_{N_{3}N_{2}}$ | 1.881E-01 | [level] |
| $k_{N_{1}N_{3}}$ | 6.202E-01 | [level]·hour^-1^ |
| $K_{N_{1}N_{3}}$ | 7.638E-01 | [level] |
| $k_{N_{2}N_{3}}$ | 2.879E+00 | [level]·hour^-1^ |
| $K_{N_{2}N_{3}}$ | 3.519E-01 | [level] |

Equations for the simplified model in Fig. 4A are as follows:

$$\begin{matrix} \frac{dI}{dt}=r\left(I_{0}-I\right) \\ \frac{dX}{dt}=\left(1-X\right)\frac{k_{Xa}\times I}{K_{Xa}+\left(1-X\right)}+\left(1-X\right)\frac{k_{Xb}\times X}{K_{Xb}+\left(1-X\right)}-X\frac{k_{Xc}}{K_{Xc}+X} \end{matrix}$$

**Table C. Mathematical kinetic parameters for the reduced model used in Fig. 4.**

| **Parameter** | **Value** | **Unit** |
| --- | --- | --- |
| *I_0_* | 8.000E-01 | [level] |
| *r* | 2.000E-01  (low temporal gradient) | [level]·hour^-1^ |
|  | 1.000E+00  (high temporal gradient) |  |
| *k_xa_* | 5.000E-01 | [level]·hour^-1^ |
| *K_xa_* | 5.000E-01 | [level] |
| *k_xb_* | 6.200E-01 | [level]·hour^-1^ |
| *K_xb_* | 1.000E-01 | [level] |
| *k_xc_* | 8.000E-01 | [level]·hour^-1^ |
| *K_xc_* | 1.000E-01 | [level] |
